# Factors associated with condom use among unmarried sexually active adolescent girls and young women (AGYW) aged 15–24 years: analysis of the Uganda Demographic Health Survey 2016

**DOI:** 10.3389/frph.2025.1590356

**Published:** 2025-09-10

**Authors:** Bruce Tukamushaba, Annette Kyomuhangi, Humphrey Atwijukiire

**Affiliations:** Department of Epidemiology and Biostatistics, School of Public Health Makerere University, Kampala, Uganda

**Keywords:** condom use, contraceptives, adolescent girls and young women, sexually transmitted infections, HIV—human immunodeficiency virus

## Abstract

**Background:**

Adolescent girls and young women (AGYW) in Uganda face a disproportionate burden of HIV and unintended pregnancies, with low condom use exacerbating these risks. Despite efforts to promote condom use, uptake remains inconsistent among this young demographic. Additionally, there is limited research on the factors influencing condom use among unmarried sexually active AGYW in Uganda. This study aimed to assess the prevalence and factors associated with condom use in this high-risk group using data from the 2016 Uganda Demographic and Health Survey (UDHS), to inform targeted interventions aimed at improving sexual health outcomes for this specific demographic.

**Methods:**

A cross-sectional analysis was conducted using secondary data from the 2016 UDHS. The study included 2,132 unmarried sexually active AGYW aged 15–24. Data were analyzed using weighted logistic regression to identify factors associated with condom use, adjusting for survey design characteristics. The outcome of interest was condom use and some of the potential predictor variables examined were; age, region, education, wealth, marital status, and exposure to family planning messages. All data processing and analysis was done using STATA v14.

**Results:**

Only 10.36% of AGYW participants reported using condoms. Factors significantly associated with condom use included region (lower use in Northern and Western regions), marital status (lower use among those ever in a union), exposure to family planning messages (higher use), and having multiple sexual partners (higher use). Recent sexual activity was also linked to increased condom use.

**Conclusions/Recommendations:**

The low prevalence of condom use highlights the urgent need for targeted interventions to address barriers such as regional disparities, limited access to sexual health education, and gender dynamics. Recommendations include implementing region-specific sexual health programs, expanding family planning messaging, and establishing youth-friendly health services. Empowering AGYW to negotiate condom use and addressing socio-economic barriers are crucial for improving sexual health outcomes.

## Introduction

Globally, adolescent girls and young women (AGYW) continue to bear a disproportionate burden of HIV. In 2019, despite making up only 10% of the population, AGYW aged 15–24 years in sub-Saharan Africa accounted for 24% of new HIV infections (1). This highlights the urgent need for innovative HIV prevention strategies to curb the epidemic within this vulnerable population (2). The vulnerability of adolescent girls and young women (AGYW) to HIV is shaped by a complex interplay of biological, behavioral, social, and structural factors (3). Proximally, the presence of sexually transmitted infections (STIs) increases HIV incidence among young women. Behaviorally, engaging in transactional sex, having multiple sexual partners, substance abuse, and inconsistent condom use elevate their risk of infection (3). Additionally, structural determinants such as parental loss and lack of access to education further contribute to HIV susceptibility (3).

Condom use plays a crucial role in preventing both sexually transmitted infections (STIs), including HIV, and unintended pregnancies, particularly among sexually active individuals (4, 5). When used consistently and correctly, male latex condoms significantly reduce the risk of HIV and other STIs (6). Epidemiological research supports their effectiveness, showing that current HIV prevalence rates would be approximately five times higher without condom use (4). Additionally, condoms provide strong protection against STIs transmitted through genital fluids, such as gonorrhea, chlamydia, and trichomoniasis, further emphasizing their importance in sexual and reproductive health (4).

Uganda has one of the highest adolescent fertility rates in sub-Saharan Africa, with approximately 24% of girls aged 15–19 having begun childbearing in 2022 (7). Additionally, Uganda has a generalized HIV epidemic, with AGYW disproportionately affected. The HIV prevalence among adolescent girls and young women (AGYW) aged 15–24 years is 3.3%, compared to 0.8% among young men (8).

Inconsistent and low use of condoms is one of the main drivers of HIV infection, other sexually transmitted infections (STIs), and unintended pregnancies, particularly among adolescent girls and young women (AGYW) who are at high risk (4). In Uganda, efforts by the government and development partners to promote condom use among AGYW include the provision of free condoms in health facilities, schools, and community centers, as well as HIV counseling and testing services, sexual health education programs, and outreach sensitization campaigns. Despite these efforts, condom use among AGYW remains low (9). This low prevalence of condom use is concerning, given the high rates of HIV and STIs among AGYW in Uganda (8).

Multiple socio-demographic and behavioral factors influence condom use among sexually active AGYW. Studies have shown that age plays a role, with older AGYW (20–24 years) more likely to use condoms compared to younger adolescents (15–19 years), potentially due to greater autonomy and awareness of reproductive health risks (10). Additionally, education level is a significant determinant, with higher levels of education associated with increased condom use due to better access to information on contraception and STI prevention (11, 12). Other factors include economic status, place of residence, number of sexual partners, religion, media exposure, partners influence and substance use (9–11, 13).

Despite the availability of condoms and ongoing efforts to promote their use, there is limited research on the factors influencing condom use among unmarried sexually active AGYW in Uganda. Existing studies often aggregate data across different age groups or marital statuses, which may obscure the unique challenges faced by this demographic. This study aims to investigate the prevalence of condom use among unmarried sexually active young women aged 15–24 years in Uganda and the factors influencing their use, utilizing data from the 2016 UDHS. By addressing these gaps, this research will inform targeted interventions aimed at improving sexual health outcomes among AGYW in Uganda.

## Methods

### Study design

This was a cross sectional study using secondary data from the 2016 Uganda Demographic Health Survey.

### Dependent (outcome variable)

The outcome variable was condom use, which was generated from the variable V312 (Current contraceptive method) in the UDHS 2016 women dataset. DHS experts generally use this variable to estimate the prevalence of condom use among women of reproductive age (12). Condom use variable was coded “yes” if young women declared condom use during the last 4 months preceding the survey, and “no” otherwise. See Table 1 for description and recoding.

**Table 1:** Description of predictor and outcome variables.

Study variable	DHS code and label	Variable type	Analysis type and recoding
Age	V013 “age in 5 years groups”	Categorical (1 “15–19”, 2 “20–24”)	Categorical
Region	V024 “region”	Categorical	Categorical (recoded to four regions: 1 “Central”, 2 “Eastern”, 3 “Northern”, 4 “Western”)
Place of residence	V025 “Place of residence”	Categorical (1 “urban”, 2 “rural”)	Categorical
Religion	V024 “Religion”	Categorical (Anglican, Catholic, Muslim, seventh day Adventist, orthodox, Pentecostal/born again/evangelical, Baptist, Jehovah's Witness, traditional, other)	Categorical (religion was recoded to 1 “anglican” 2 “catholic” 3 “muslim” 4 “pentecostal” 5 “others”)
Highest level of education	V106 “highest level of education”	Categorical (0 “No education,” 1 “Primary,” 2 “Secondary,” 3 “Higher”)	Categorical
Wealth index combined	V190 “wealth index combined” It was constructed using principal component analysis (PCA) based on data on household ownership of durable assets (such as a radio, bicycle, mobile phone, or television), dwelling characteristics (such as floor, roof, and wall materials), and access to utilities (such as electricity, water source, and type of sanitation facility). The PCA generated a wealth score for each household, and based on these scores, households were ranked and divided into five wealth quintiles	Categorical (1 “lowest,” 2 “second,” 3 “middle,” 4 “fourth,” 5 “highest”)	Categorical (Lowest, Second, Middle, Fourth, Highest)
Ever in union	V501 “Marital status”	Categorical (0 “Never in union”, 1 “Married”, 2 “Living with partner”, 3 “Widowed”, 4 “Divorced”, 5 “No longer living together/separated”)	Categorical (recoded to v501_cat “v501_ever_in_union”: 1 “Yes”, 0 “No”) For this study, currently married individuals were excluded during initial data filtering. The remaining categories were recoded into a binary variable: “No” for those who had never been in a union (never in union), and “Yes” for those who had ever been in a union but were no longer currently married (widowed, divorced, or separated).
Currently working	V714 “respondent currently working”	Categorical (0 “No”, 1 “Yes”)	Categorical
FP message exposure	Generated from variables: v384a “listening FP on radio”, v384b “listening FP on TV”, v384c “heard family planning in newspaper/magazine”, v384d “heard family planning by text messages”	Categorical (each coded as 0 “No”, 1 “Yes”)	Categorical (recoded to FP message exposure for every individual who has ever received FP message via any media: 1 “Yes” and 0 “No”)
FP knowledge	V301 “Knows any method”	Categorical (0 “knows no method” 3 “knows modern method”)	Categorical (recoded to v301_cat: 1 “Yes”, 0 “No”)
Knows ovulatory cycle	V217 “knowledge of ovulatory cycle”	Categorical 1 “during her period”, 2 “after period ended”, 3 “middle of the cycle”, 4 “before period begins”, 5 “at any time”, 6 “other” 8 “don't know”	Categorical (recoded v217_cat “knows ovulatory cycle”: 1 “Yes”, 0 “No”)
Sexual partners, in last 12 months	V766b “Number of sex partners, including spouse, in last 12 months”	Continuous (0–95)	Categorical (recoded to sexual partners, in last 12 months: 0 “No partner”, 1 “1 partner”, 2 “2 or more partners”)
Recent sexual activity	V536 “Recent sexual activity”	Categorical (“Active in last 4 weeks”, 2 “Not active in last 4 weeks—postpartum abstinence”, 3 “Not active in last 4 weeks—not postpartum abstinence”)	Categorical (recoded to v536_act: 1 “Active in last 4 weeks”, 2 “Not active in last 4 weeks”)
Ever tested for HIV	V781 “ ever tested for HIV”	Categorical (0 “No”, 1 “Yes”)	Categorical
Ever heard of STI	V750 “ever of STI”	Categorical (0 “No”, 1 “Yes”)	Categorical
Uses cigarette or tobacco	V463z “does not use cigarettes and tobacco”	Categorical (0 “no”, 1 “yes, smokes nothing”	Categorical (0 “No” 1 “Yes”)
Condom use	V312 “current contraceptive method”	Categorical [not using, pills, iud, injections, male condom, female sterilization, male sterilization, periodic abstinence, withdrawal, other traditional, implants/norplant, lactational amenorrhea (lam), female condo, emergency contraception, other modern method, standard days method (sdm)]	Categorical (condom use “uses condom as a form of contraception”: 1 “yes” 0 “No”)
No			
Yes			

### Potential predictor variables

The study examines several predictor variables associated with condom use among unmarried sexually active young women aged 15–24 years in Uganda. Age is categorized into two groups: (15–19) and (20–24). Region is divided into (Central, Eastern, Northern, and Western). Place of residence is classified as (Urban and Rural). Religion includes (Anglican, Catholic, Muslim, Pentecostal/Born Again, and Others). Highest level of education is categorized into (No education, Primary, Secondary, and Higher). Wealth index is classified into five levels: (Lowest, Second, Middle, Fourth, and Highest). Ever in union refers to marital status and is divided into (No and Yes). Currently working distinguishes between (No and Yes). Family planning (FP) message exposure is classified as (No and Yes), while FP knowledge is also categorized as (No and Yes). Knowledge of the ovulatory cycle is grouped into (No and Yes). Sexual partners in the last 12 months include (No partner, 1 partner, and 2 or more partners). Recent sexual activity is categorized into (Active last 4 weeks and Not active last 4 weeks). HIV testing history is classified as (No and Yes). Awareness of sexually transmitted infections (STIs) is categorized as (No and Yes). Finally, cigarette or tobacco use is classified into (No and Yes). See Table 1 for full description and re-categorization of the above predictor variables.

### Sample design

The 2016 UDHS used the sampling frame of the Uganda National Population and Housing Census (NPHC), conducted in 2014. The census frame is a complete list of all census enumeration areas (EAs) created for the 2014 NPHC. During the NPHC Uganda was divided administratively into 112 districts, these were grouped into 15 regions for this survey. Three special areas, the Lake Victoria islands, the mountain districts, and greater Kampala, were also considered.

The sample was stratified and selected in two stages. In the first stage, 697 EAs (162 and 535 EAs in urban and rural areas respectively) were selected from the 2014 Uganda NPHC. The second stage of sampling constituted households. In each of the 696 accessible selected EAs, a household listing was conducted, and to reduce the workload, any large EA with more than 300 households was segmented for the 2016 UDHS.

A total of 20,880 households were selected for the 2016 UDHS, with 30 households randomly chosen from each enumeration area (EA) or EA segment to ensure representativeness.

### Sampling size calculation

A representative sample of 20,880 households was randomly selected for the 2016 Uganda DHS, with 19,088 eligible women being identified. Interviews were completed with 18,506 (97.0%) women and of 18,506 women, 8,058 were adolescent girls and young women aged 15–24. All adolescent girls who were married or living with partners (*n* = 3,328) were excluded from the analysis. Additionally, adolescent girls and young women who reported not being sexually active (*n* = 2,597) were also removed. One observation with missing data for the variable recent sexual activity was dropped (*n* = 1). After these exclusions, the final sample of adolescent girls and young women who met the inclusion criteria for the analysis consisted of 2,132 participants.

### Inclusion and exclusion criteria

#### Inclusion criteria

All Ugandan adolescent girls and young women (15–24) years who were either permanent residents of the selected households or visitors who stayed in the household the night before the survey were eligible to participate.

#### Exclusion criteria

Any adolescent girls and young women (15–24) who are married, living with partners and have never had sex were excluded from the analysis.

Any adolescent girls and young women (15–24) with missing data were also exclude from the analysis.

### Data management and analysis

#### Data management

The 2016 Uganda Demographic and Health Survey (UDHS) dataset was used for this study, specifically the women's dataset (UGIR7AFL.DTA) for women aged 15–49. The Standard Recode Manual for DHS7 was consulted to understand how the variables were collected and coded. This manual provided guidance on the structure of the dataset, the meaning of variable codes, and the methodology used during data collection.

To ensure data completeness and accuracy, code booking and tabulation of the variables of interest were conducted. This process involved checking the frequency and distribution of each variable to identify any missing or inconsistent data. The variables were categorized as either continuous or categorical and their respective ranges or categories were documented. This step was crucial for understanding the dataset and preparing it for analysis. Some of the variables of interest were recoded and renamed before analysis.

All outcome and potential predictor variables were checked for duplicates and assessed for missing values prior to analysis. Only variables with complete data were included in the final analysis. Data processing and analysis were conducted using STATA version 14.

### Univariable analysis

The analysis began by declaring the survey design characteristics using the command “svyset” to account for complex survey design [weighting (wgt), clustering (v021), stratification (v023) were used]. Unweighted frequencies and percentages for the different categories of the variables were obtained through tabulation. Weighted percentages were then calculated by adding the “svy” prefix to the tabulation command, ensuring that the results were representative of the population.

### Bivariable analysis

The bivariable analysis aimed to assess the association between each independent variable and the outcome variable (condom use). Weighted cross-tabulations of the outcome variable and each independent variable were conducted to obtain weighted percentages of women who used condoms as form of contraceptive.

Binary logistic regression was used for bivariable analysis to obtain crude odds ratios (CORs), 95% confidence intervals (CIs), and *p*-values. The “svy” prefix was used to account for the survey design. A significance threshold of *p* < 0.25 was used at this stage to ensure that potentially important variables were not excluded prematurely. This threshold allowed for the inclusion of variables that might show significance when adjusted for other factors in the multivariable analysis.

The bivariable analysis provided initial insights into the factors associated with condom use, guiding the selection of variables for the multivariable model.

### Multivariable analysis

All independent variables that were statistically significant in the bivariable analysis were assessed for multicollinearity prior to inclusion in the multivariable model. Multicollinearity was examined using the “correlate” command in STATA, and a correlation coefficient of 0.4 or higher was considered indicative of high multicollinearity. In cases where two or more variables were highly correlated, one variable was excluded from the multivariable analysis based on prior literature and the strength of association observed in the bivariable analysis.

The multivariable analysis aimed to identify the independent predictors of condom use while controlling for potential confounding factors. Binary logistic regression was used, and the backward elimination method was employed to build the final model. Variables with the least significance (based on *p* < 0.05) were removed step-by-step. This method ensured that only the most significant predictors remained in the final model.

The goodness of fit of the final model was tested using the “estat gof” command, which yielded a Prob > *F* = 0.7132, indicating that the model fit the data well. The “svy” prefix was used throughout the regression analysis to account for the survey design.

## Results

### Background characteristics of unmarried sexually active adolescent girls and young women (15–24)

Table 2 presents the socio-demographic characteristics of unmarried sexually active adolescent girls and young women aged 15–24 years in Uganda. The sample size was 2,132; with 50.75% aged 15–19 and 49.25% aged 20–24. Geographically, 36.38% resided in the Central region, 27.56% in the Eastern region, 15.93% in the Northern region, and 20.13% in the Western region. The majority (67.71%) lived in rural areas. By religious affiliation, 31.36% were Anglican, 38.29% Catholic, 14.10% Muslim, and 13.87% Pentecostal/Born Again. Educationally, 48.82% had primary education, 38.78% secondary, and 11.16% higher education. Wealth distribution showed 34.19% in the highest wealth quintile and 12.28% in the lowest. Additionally, 75.85% had never been in a union, 63.79% were currently working, and 70.73% had exposure to family planning (FP) messages. Knowledge of FP was nearly universal (99.35%), and 85.69% knew their ovulatory cycle. Regarding sexual behavior, 69.52% had one sexual partner in the last 12 months, and 20.16% were sexually active in the last 4 weeks. HIV testing was reported by 82.91%, and 99.86% had heard of STIs. Only 10.36% reported using condoms during their sexual intercourse.

**Table 2:** The socio-demographic characteristics of single sexually active adolescent girls and young women (15–24).

Variable	Un weighted number	Un weighted percent	Weighted percent
Age			
15–19	1,082	50.75	50.46
20–24	1,050	49.25	49.54
Region			
Central	652	30.58	36.38
Eastern	631	29.60	27.56
Northern	395	18.53	15.93
Western	454	21.29	20.13
Place of residence			
Urban	668	31.33	32.29
Rural	1,464	68.67	67.71
Religion			
Anglican	677	31.75	31.36
Catholic	834	39.12	38.29
Muslim	282	13.23	14.10
Pentecostal/born again	287	13.46	13.87
Others	52	2.44	2.38
Highest level of education			
No education	38	1.78	1.24
Primary	1,079	50.61	48.82
Secondary	784	36.77	38.78
Higher	231	10.83	11.16
Wealth index combined			
Lowest	311	14.59	12.28
Second	356	16.70	15.74
Middle	355	16.65	15.66
Fourth	437	20.50	22.13
Highest	673	31.57	34.19
Ever in union			
No	1,632	76.55	75.85
Yes	500	23.45	24.15
Currently working			
No	763	35.79	36.21
Yes	1,369	64.21	63.79
FP message exposure			
No	631	29.60	29.27
Yes	1,501	70.40	70.73
FP knowledge			
No	19	0.89	0.65
Yes	2,113	99.11	99.35
Knows ovulatory cycle			
No	295	13.84	14.31
Yes	1,837	86.16	85.69
Sexual partners, in last 12 months			
No partner	537	25.19	24.86
1 partner	1,479	69.37	69.52
2 or more partners	116	5.44	5.63
Recent sexual activity			
Active last 4 weeks	438	20.54	20.16
Not active last 4 weeks	1,684	79.46	79.84
Ever tested for HIV			
No	366	17.17	17.09
Yes	1,766	82.83	82.91
Ever heard of STI			
No	4	0.19	0.14
Yes	2,128	99.81	99.86
Uses cigarette or tobacco			
No	17	0.80	0.57
Yes	2,115	99.20	99.43
Condom use			
No	1,922	90.15	89.64
Yes	210	9.85	10.36

∑*n* = 2,132.

### Prevalence of condom use by socio-demographic characteristics of unmarried sexually active adolescent girls and young women (15–24)

Table 3 shows the prevalence of condom use and crude odds ratios (CORs) by socio-demographic characteristics. Condom use was significantly lower in the Northern (0.84%) and Western (1.19%) regions compared to the Central region (4.64%) (*p* < 0.01). Rural residents had lower condom use (5.98%) compared to urban residents (4.38%) (*p* < 0.01). Women with primary education had significantly lower condom use (2.65%) compared to those with higher education (1.98%) (*p* < 0.001). Women who had ever been in a union had lower condom use (1.14%) compared to those who had never been in a union (9.22%) (*p* < 0.01). Exposure to FP messages was associated with higher condom use (8.4%) compared to no exposure (1.96%) (*p* < 0.01). Women with one sexual partner (8.96%) or multiple partners (1.00%) in the last 12 months had significantly higher condom use compared to those with no partner (0.39%) (*p* < 0.001). Recent sexual activity in the last 4 weeks was associated with higher condom use (4.00%) compared to no recent activity (6.36%) (*p* < 0.001).

**Table 3:** Prevalence of condom use by socio-demographic characteristics of single sexually active adolescent girls and young women (15–24) and the crude odds ratios (CORs).

Variable	Condom use		Crude OR (95% CI)
	No (%)	Yes (%)	
Age			
15–19	977 (44.94)	120 (5.53)	1
20–24	972 (44.71)	105 (4.83)	0.878 (0.634–1.216)
Region			
Central	690 (31.74)	101 (4.64)	1
Eastern	519 (23.88)	80 (3.68)	1.054 (0.687–1.616)
Northern	328 (15.08)	18 (0.84)	0.382 (0.220–0.664)**
Western	412 (18.94)	26 (1.19)	0.431 (0.258–0.717)**
Place of residence			
Urban	607 (27.91)	95 (4.38)	1
Rural	1,343 (61.74)	130 (5.98)	0.617 (0.428–0.890)**
Religion			
Anglican	612 (28.16)	70 (3.20)	1
Catholic	751 (34.55)	81 (3.75)	0.954 (0.627–1.450)
Muslim	270 (12.44)	36 (1.66)	1.172 (0.670–2.054)
Pentecostal/born again	269 (12.36)	33 (1.51)	1.075 (0.606–1.905)
Others	46 (2.14)	5 (0.24)	0.993 (0.391–2.523)
Highest level of education			
No education	27 (1.24)	0	(empty)
Primary	1,004 (46.17)	58 (2.65)	0.266 (0.154–0.457)***
Secondary	719 (33.06)	124 (5.72)	0.802 (0.531–1.210)
Higher	200 (9.18)	43 (1.98)	1
Wealth index combined		225 (10.36)	
Lowest	247 (11.37)	20 (0.91)	1
Second	321 (14.78)	21 (0.96)	0.812 (0.431–1.534)
Middle	306 (14.06)	35 (1.60)	1.427 (0.692–2.945)
Fourth	431 (19.82)	50 (2.31)	1.460 (0.720–2.959)
Highest	644 (29.61)	100 (4.58)	1.938 (0.994–3.777)
Ever in union			
No	1,449 (66.66)	201 (9.22)	1
Yes	501 (23.02)	25 (1.14)	0.357 (0.199–0.640)**
Currently working			
No	699 (32.13)	89 (4.08)	1
Yes	1,251 (57.51)	136 (6.28)	0.860 (0.624–1.185)
FP message exposure			
No	594 (27.31)	42 (1.96)	1
Yes	1,356 (62.34)	182 (8.40)	1.879 (1.257–2.808)**
Knows ovulatory cycle			
No	282 (12.97)	29 (1.33)	1
Yes	1,668 (76.67)	196 (9.02)	1.144 (0.692–1.892)
Sexual partners, in last 12 months			
No partner	532 (2,446)	8 (0.39)	1
1 partner	1,317 (60.56)	195 (8.96)	9.235 (4.150–20.553)***
2 or more partners	101 (4.63)	22 (1.00)	13.540 (5.075–36.121)***
Recent sexual activity			
Active last 4 weeks	351 (16.6)	87 (4.00)	1
Not active last weeks	1,598 (73.48)	138 (6.36)	0.350 (0.247–0.496)***
Ever tested for HIV			
No	328 (15.08)	44 (2.001)	1
Yes	16.22 (74.57)	181 (8.35)	0.840 (0.552–1.279)

* *p* < 0.05.

** *p* < 0.01.

*** *p* < 0.001.

### Factors associated with condom use among unmarried sexually active adolescent girls and young women (15–24)

Table 4 presents the adjusted odds ratios (AORs) from the multivariable logistic regression. Women in the Northern (AOR = 0.512, 95% CI: 0.281–0.930) and Western (AOR = 0.489, 95% CI: 0.283–0.843) regions had significantly lower odds of condom use compared to the Central region (*p* < 0.01). Women who had ever been in a union had lower odds of condom use (AOR = 0.346, 95% CI: 0.188–0.634) compared to those who had never been in a union (*p* < 0.01). Exposure to FP messages was associated with higher odds of condom use (AOR = 1.669, 95% CI: 1.108–2.512) (*p* < 0.01). Women with one sexual partner (AOR = 8.102, 95% CI: 3.607–18.201) or multiple partners (AOR = 9.961, 95% CI: 3.668–27.051) in the last 12 months had significantly higher odds of condom use compared to those with no partner (*p* < 0.001). Not being sexually activity in the last 4 weeks was associated with lower odds of condom use (AOR = 0.504, 95% CI: 0.354-0.719) compared to being sexually active in the last 4 weeks (*p* < 0.001).

**Table 4:** Weighted modified logistic regression estimates for condom use by socio-demographic characteristics of single sexually active adolescent girls and young women (15–24) and the adjusted odds ratios (AORs).

Variable	Condom use		Crude OR (95% CI)	Adjusted OR (95% CI)
	No (%)	Yes (%)		
Region				
Central	690 (31.74)	101 (4.64)	1	1
Eastern	519 (23.88)	80 (3.68)	1.054 (0.687–1.616)	1.072 (0.680–1.691)
Northern	328 (15.08)	18 (0.84)	0.382 (0.220–0.664)**	0.512 (0.281–0.930)**
Western	412 (18.94)	26 (1.19)	0.431 (0.258–0.717)**	0.489 (0.283–0.843)**
Place of residence				
Urban	607 (27.91)	95 (4.38)	1	1
Rural	1,343 (61.74)	130 (5.98)	0.617 (0.428–0.890)**	0.746 (0.508–1.095)
Ever in union				
No	1,449 (66.66)	201 (9.22)	1	1
Yes	501 (23.02)	25 (1.14)	0.357 (0.199–0.640)**	0.346 (0.188–0.634)**
FP message exposure				
No	594 (27.31)	42 (1.96)	1	1
Yes	1,356 (62.34)	182 (0.84)	1.879 (1.257–2.808)**	1.669 (1.108–2.512)**
Sexual partners, in last 12 months				
No partner	532 (2,446)	8 (0.39)	1	1
1 partner	1,317 (60.56)	195 (8.96)	9.235 (4.150–20.553)***	8.102 (3.607–18.201)***
2 or more partners	101 (4.63)	22 (1.00)	13.540 (5.075–36.121)***	9.961 (3.668–27.051)***
Recent sexual activity				
Active last 4 weeks	351 (16.6)	87 (4.00)	1	1
Not active last weeks	1,598 (73.48)	138 (6.36)	0.350 (0.247–0.496)***	0.504 (0.354–0.719)***

Logistic model for condom use, goodness-of-fit test “estat gof”. *F*_(9,584)_ = 0.70, Prob > *F* = 0.7132.

* *p* < 0.05.

** *p* < 0.01.

*** *p* < 0.001.

## Discussion

This study aimed to determine the factors associated with condom use among unmarried sexually active young women aged 15–24 years in Uganda, using data from the 2016 Uganda Demographic and Health Survey (UDHS). The findings revealed a low overall prevalence of condom use among this high-risk population, with notable differences across regions, relationship histories, and behavioral factors. Condom use was more likely among women recently sexually active, those with multiple sexual partners, and those exposed to family planning messages. Conversely, lower condom use was observed among women who had ever been in a union and those living in certain regions. These findings highlight key behavioral and contextual factors that must be addressed to improve protective sexual practices among young Ugandan women.

The analysis revealed that only 10.36% of the participants reported using condoms, highlighting a significant gap in the adoption of protective sexual health practices among this high-risk group. This finding is lower than the prevalence reported in other sub-Saharan African countries, such as Malawi, where 18.5% of young women reported condom use 4 months prior to the survey (14), and Haiti, where 15.4% of sexually active young women reported condom use (11). The low prevalence of condom use in Uganda underscores the urgent need for targeted interventions to address the barriers to condom use among young women, particularly in the context of high HIV prevalence and unintended pregnancies (15).

The study found significant regional disparities in condom use, with lower usage in the Northern and Western regions compared to the Central region. This aligns with findings from Tanzania, where regional variations in condom use were attributed to differences in access to sexual health services and cultural norms (16). The Northern and Western regions of Uganda may face similar challenges, such as limited healthcare infrastructure and conservative cultural attitudes toward condom use. These findings underscore the need for region-specific interventions to address barriers to condom use, particularly in underserved areas.

Women who had ever been in a union had lower odds of condom use, consistent with studies from Malawi and Ethiopia that found married individuals are less likely to use condoms due to perceived lower STI risk within marital relationships (14, 17, 18). However, this perception can increase vulnerability to STIs, particularly in non-monogamous relationships. Interventions should focus on promoting condom use within marital relationships, emphasizing the importance of dual protection against STIs and unintended pregnancies.

Exposure to FP messages was associated with higher condom use, consistent with findings from Nigeria and Malawi (10, 12), that emphasize the role FP messages and media exposure in promoting awareness of the benefits of condom use and provide information on how to access condoms. Expanding FP messaging through media and community outreach programs could further enhance condom use among young women.

Women with one or more sexual partners in the last 12 months had significantly higher odds of condom use, consistent with studies from Brazil, Haiti and Uganda (4, 11, 19). This suggests that women with multiple partners may be more aware of the risks associated with unprotected sex. However, the low overall prevalence of condom use (10.36%) indicates that many young women remain at risk. Interventions should focus on promoting consistent condom use, particularly among women with multiple partners.

Recent sexual activity in the last 4 weeks was associated with higher condom use, consistent with findings from Uganda (9), that found a correlation between sexual contact and condom use. This may reflect increased awareness of the need for protection during recent sexual encounters. However, the low prevalence (4%) of condom use among women who were recently sexually active suggests that many women may not perceive the need for consistent condom use. Public health campaigns should emphasize the importance of consistent condom use, regardless of recent sexual activity.

### Study strengths

This study utilized data from the 2016 Uganda Demographic and Health Survey (UDHS), which is a nationally representative dataset. This ensures that the findings are generalizable to the broader population of unmarried sexually active young women aged 15–24 years in Uganda.

The study included a large sample size of 2,132 participants, which enhances the statistical power and reliability of the results. This allows for more analyses that are robust and the identification of significant associations between variables.

The study employed a multivariable logistic regression model will adjusting for survey design characteristics, which offers more accurate estimates and allows accurate understanding of the predictors of condom use.

By focusing on unmarried sexually active young women, the study addresses a high-risk group that is often overlooked in broader sexual health research. This targeted approach provides valuable insights for designing interventions tailored to this demographic.

### Study limitations

The study is based on cross-sectional data, which limits the ability to establish causal relationships between the variables.

The data on condom use and sexual behavior were self-reported, which may be subject to social desirability bias. Participants may underreport risky sexual behaviors or over report condom use, leading to potential inaccuracies in the findings.

The study relies solely on quantitative data, which does not provide deeper insights into the barriers and facilitators of condom use among young women in Uganda.

The study focuses exclusively on young women and does not include the perspectives of their male partners. Given the role of gender dynamics in sexual decision-making, understanding male attitudes and behaviors could provide a more comprehensive picture of condom use.

## Conclusion

This study highlights the persistently low uptake of condoms among unmarried sexually active adolescent girls and young women (AGYW) in Uganda, underscoring a critical public health concern. The analysis identified key behavioral and contextual factors influencing condom use, suggesting the need for targeted, context-specific interventions. Enhancing access to comprehensive sexual and reproductive health education, particularly through mass media, schools, and community outreach, can play a pivotal role in increasing awareness and acceptance of condom use. Moreover, empowering AGYW with the skills and agency to negotiate safer sexual practices is essential to reducing the risks of HIV, other sexually transmitted infections, and unintended pregnancies. Policymakers and program implementers must prioritize inclusive, youth-friendly strategies that address the unique barriers faced by this population, with particular attention to regional and sociocultural disparities. Strengthening these efforts is vital for advancing sexual and reproductive health outcomes and promoting the well-being of Uganda's young women.

## Recommendations

Based on the findings of this study, the following recommendations are proposed to improve condom use among unmarried sexually active AGYW in Uganda:

Tailored sexual health programs should be implemented in the Northern and Western regions, where condom use is significantly lower. These programs should address cultural barriers and improve access to sexual health services. Furthermore, qualitative studies in these regions are essential to gain deeper insights into the specific challenges and barriers influencing condom use, thereby informing more effective, context-sensitive interventions.

Expand the reach of family planning messages through radio, television, and social media to increase awareness of condom use. Messages should be culturally sensitive and address misconceptions about condoms, such as discomfort or reduced sexual pleasure.

Finally, future researchers should conduct longitudinal studies to understand the causal relationships between socio-demographic factors and condom use. Additionally, incorporate qualitative research to explore the barriers and facilitators to condom use and also explore the role of gender and partner dynamics.

## Data Availability

Publicly available datasets were analyzed in this study. This data can be found here: https://dhsprogram.com/data/dataset/Uganda_Standard-DHS_2016.cfm?flag=0.
